# Metastatic Myoepithelial Carcinoma Ex Pleomorphic Adenoma of the Sublingual Salivary Gland

**DOI:** 10.7759/cureus.39912

**Published:** 2023-06-03

**Authors:** Ângela T Ferreira, Joana Gonçalves, Andreia Ferreira, José Ricardo Brandão, Rute Saleiro

**Affiliations:** 1 Maxillofacial Surgery Department, Centro Hospitalar Universitário de Santo António, Porto, PRT; 2 Pathology, Centro Hospitalar Universitário de Santo António, Porto, PRT

**Keywords:** basic fibroblast growth factor, lung metastasis, myoepithelial carcinoma, salivary gland, carcinoma ex pleomorphic adenoma

## Abstract

Myoepithelial carcinoma ex pleomorphic adenoma is a very rare malignant neoplasm of the salivary gland. Owing to its rarity, its clinical features and treatment are not well characterized. We describe a case of a patient who was referred to our department with a six-month history of a bulge on the right side of the floor of the mouth and a submandibular mass with progressive enlargement. The mass was resected, and an elective level I neck dissection was performed. Histological examination revealed myoepithelial carcinoma ex pleomorphic adenoma of the sublingual salivary gland. Thoracic computed tomography and biopsy revealed lung metastases. The patient died two years after the diagnosis.

## Introduction

Malignant tumors of the salivary gland are histologically diverse and uncommon. The World Health Organization classification of head and neck tumors recognizes more than 20 different types of these neoplasms, with one of them being myoepithelial carcinoma (MECA) [1,2]. This rare and aggressive tumor has a relatively low incidence and accounts for less than 1% of all malignant salivary gland tumors. The most frequent location is the parotid gland, although other salivary glands may be affected [2,3]. Its poorly defined diagnostic criteria make it very challenging to identify. The histological diagnosis of myoepithelial carcinoma ex pleomorphic adenoma is not straightforward due to its morphological heterogeneity [4]. Histologically, MECA is a tumor in which the myoepithelial cells are prone to penetrate the tumor borders. If the infiltrative pattern and cell atypia are clearly seen, classification as a high-grade tumor is obvious. However, MECA tends to appear without aggressive features, which may lead to delay or failure in the diagnosis [4]. These tumors usually appear in the sixth and seventh decades of life [3]. MECA can develop from pre-existing pleomorphic adenomas, which are the most common, or from benign myoepitheliomas; it can also arise de novo [2,5,6].

MECA has been described as the second most common histologic type of carcinoma, ex pleomorphic adenoma, after salivary duct carcinoma [3]. Carcinoma ex pleomorphic adenoma develops from pleomorphic adenomas, and this tumor type accounts for roughly 3.6% of all salivary gland tumors and 5%-15% of salivary gland malignancies [1,4]. These tumors usually present as a mass whose growth in a pre-existing nodule (recurrent pleomorphic adenoma in 12% of cases) is painless and very fast [1,3]. The risk of pleomorphic adenoma transformation into carcinoma after diagnosis is 1.5% within five years and up to 10% within 15 years [7].

Here, we present a case of MECA ex pleomorphic adenoma in which, despite treatment with chemotherapy and radical surgery, this was not enough to heal the malignant neoplasia.

## Case presentation

In the current case, a 55-year-old woman presented to the emergency department with a six-month history of progressive asymptomatic bulging on the right side of the floor of the mouth and a right submandibular mass. She reported a weight loss of 4-5 kg in the previous three months and recent submandibular pain and dysphagia.

Four months before, she was evaluated at a minor hospital. Computed tomography (CT) revealed a hypodense lesion measuring 4.3 × 2.9 cm with small-calcified areas. Fine-needle aspiration cytology (FNAC) of the submandibular mass revealed cellular aspirate from a cystic source, and histology showed mucinous cells from a minor salivary gland with areas of atrophy, focal interstitial fibrosis and nests of mild interstitial inflammatory lymphocytic infiltrate.

**Figure 1:**
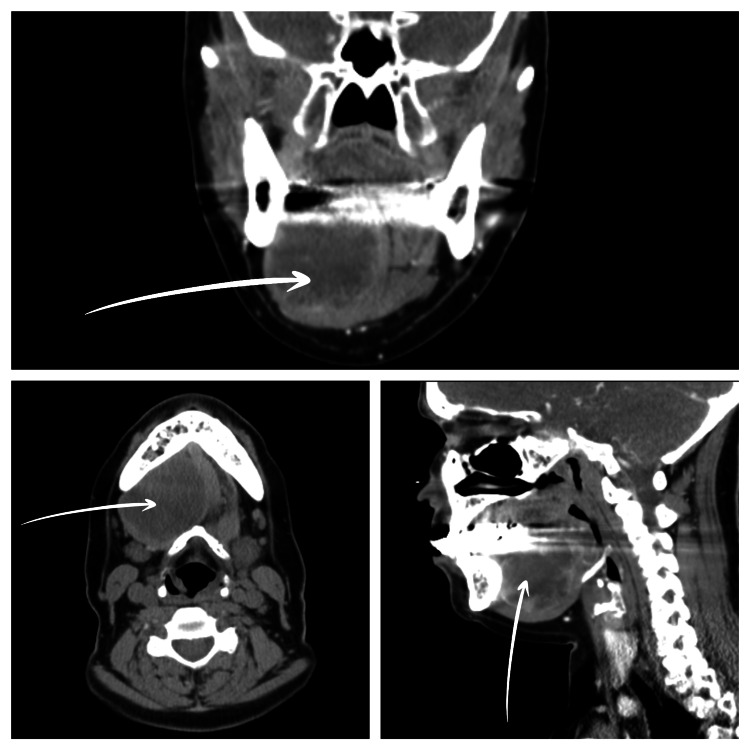
Contrast-enhanced CT images show a right-side expansive lesion centered on the floor of the mouth. Upper: coronal. Lower left: axial. Lower right: sagittal

**Figure 2:**
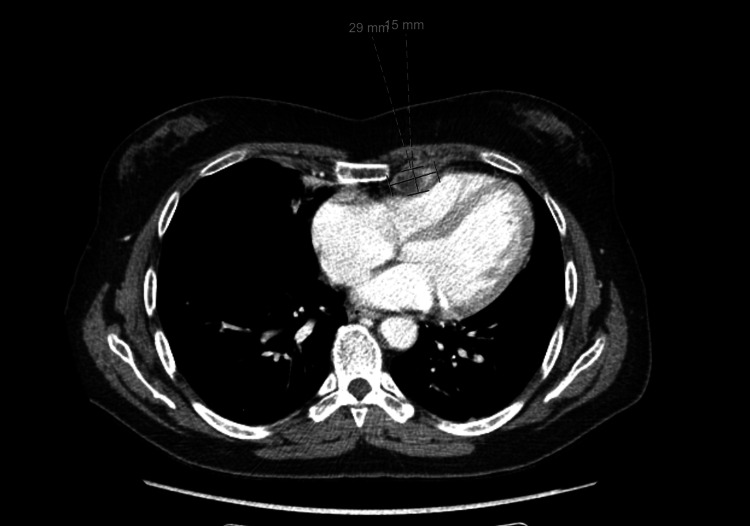
Axial CT showing necrotic-appearing lymph nodes in the left internal mammary chain

Upon palpation, the bulge on the floor of the patient’s mouth measured around 5 cm and mimicked the submandibular salivary gland. Cervical adenopathy was not palpated, and there were no changes in motion or sensibility. The patient underwent a new neck CT scan, which revealed a right-side expansive lesion centered on the floor of the mouth (Figure 1). The lesion was 4.8 × 4 cm in size, and it was rounded but without well-defined margins. A presumptive diagnosis of malignancy was made based on the aggressive appearance, despite the lack of significant cervical adenopathy. The parotid and submandibular salivary glands had normal morphology. In the thoracic CT, two necrotic-appearing lymph nodes in the left internal mammary chain were suspicious of malignancy (Figure 2).

**Figure 3:**
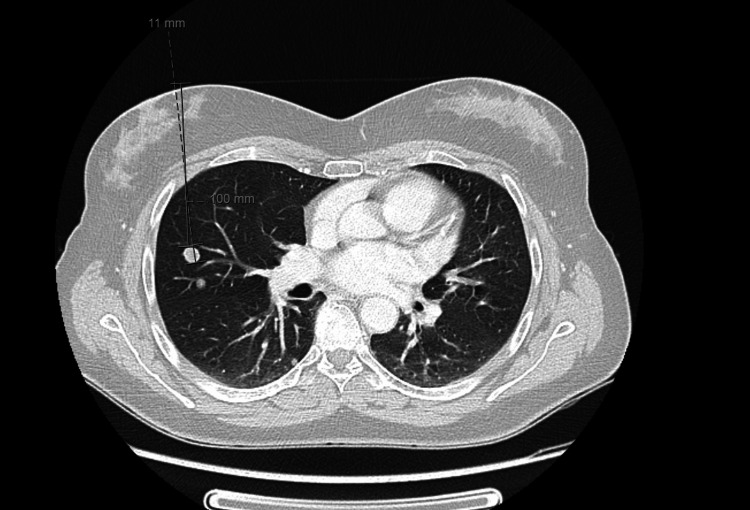
Thoracic CT with multiple lung metastases

Multiple bilateral lung nodules, as well as subpleural nodules with aleatory distribution, were also identified, suggesting lung metastasis (Figure 3).

**Figure 4:**
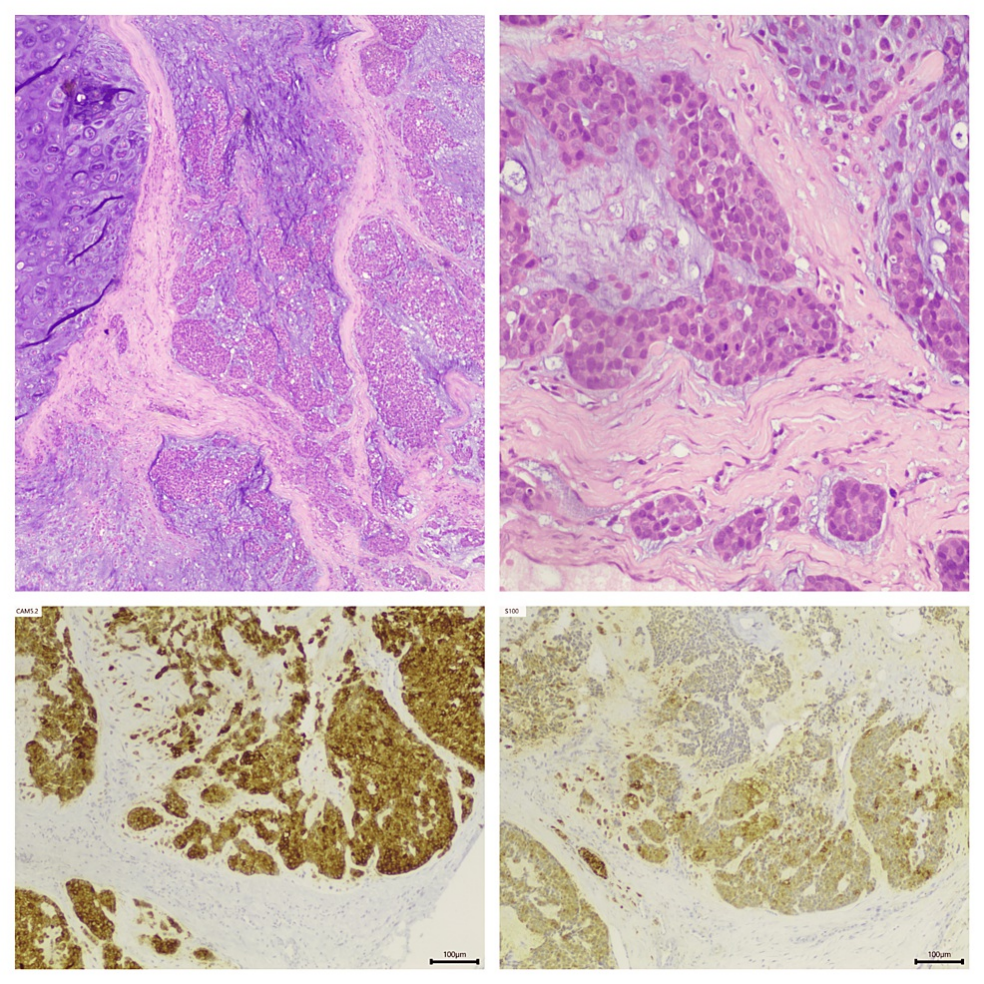
Upper: histological findings of the tumor stained with hematoxylin and eosin (H&E). Lower: immunohistochemical staining. Photo courtesy: Dr. José Ricardo Brandão Upper left: areas of pleomorphic adenoma with foci of myoepithelial carcinoma (H&E). Upper right: pleomorphic hyperchromatic nuclei with occasional prominent mitoses (H&E). Lower left: tumor cells are positive for CAM 5.2. Lower right: tumor cells are positive for S100

At that point, the decision was made to perform an excision of the tumor and level I neck dissection with the patient under general anesthesia. A pathological diagnosis of chronic sialadenitis of the submandibular gland and myoepithelial carcinoma ex pleomorphic adenoma of the sublingual salivary gland was made. Immunohistochemical staining showed the tumor was positive for S-100 and CAM 5.2 (Figure 4). 

Additional biopsies of necrotic thoracic adenopathy in the left internal mammary chain and lung nodules were performed, and histology revealed metastatic MECA. After discussion in a multidisciplinary Head and Neck Oncology consultation, a positron emission tomography scan was requested, and it revealed [18F] FDG enhancements in the floor of the mouth, lung, and mediastinal adenopathies. Fluorescence in situ hybridization testing for human epidermal growth factor receptor 2 (HER2) was negative. Foundation One study with target mutations was also requested, with results showing mutations in the AT-rich interactive domain-containing protein 1A (ARID1A) gene, MYC, rapamycin-insensitive companion of mTOR (RICTOR), and RAD21. There was fibroblast growth factor 10 (FGF10) amplification. Owing to scarce information about the treatment of this rare type of tumor and reports of chemotherapy resistance caused by RAD21 amplification, the patient was treated with lenvatinib off-label at 24 mg/d. Four months later, she started cytotoxic chemotherapy with docetaxel to control the progression of the disease. She completed six cycles with an initial excellent response; however, chest CT imaging showed lung disease progression.

The patient was followed up at the hospital in her area of residence and received palliative care until her death.

## Discussion

Carcinoma ex pleomorphic adenoma is a very rare salivary gland tumor, and MECA is also very uncommon [4,6]. We describe this type of salivary gland neoplasia in a middle-aged woman who rapidly developed signs and symptoms of metastasis. The patient presented with a six-month history of clinical signs of neoplasia in the submandibular location, and lung metastasis was immediately discovered on thoracic CT.

Preoperative diagnosis is far from accurate in myoepithelial carcinoma ex pleomorphic adenoma [4,8]. There are no characteristic or specific signs and symptoms that distinguish this type of tumor from other benign or malignant tumors of the salivary glands. Initial CT imaging showed a right expansive lesion centered on the floor of the mouth that was rounded but without well-defined margins; this lesion was heterogeneous and mainly hypodense, and it had multiple septations with focal calcifications and hyperintense peripheral thickness. Ranula was included in the differential diagnosis, but enhancement of the wall of the mass and septations, no fat densification, and a painless mass did not support saliva retention. Primary neoplasia with cystic components could not be excluded.

The FNAC of malignant salivary gland tumors is ambiguous. Its sensitivity is around 29%-54% in the diagnosis of carcinoma ex pleomorphic adenoma [8]. In our patient, FNAC could not identify malignant cells either, and the etiology was uncertain.

Postoperatively, the histology of myoepithelial carcinoma ex pleomorphic adenoma is also challenging because of its heterogeneity [2,4]. We found an encapsulated, multinodular neoplasia with a chondromyxoid stroma. It was chiefly composed of an epithelioid pattern of myoepithelial cells, and it displayed pleomorphism, high mitotic activity, and necrotic centers. There was no invasion of glandular residual stroma observed; however, findings were suggestive of perivascular invasion. Immunohistochemical analysis was positive for S-100 protein and CAM 5.2. Histologically, MECA presents with variable morphologic features, but myoepithelial cells are usually dominant and monotonous, and the pattern of growth is invasive [4,6]. The differential diagnosis of these tumors is of utmost importance because their significance and prognoses vary. MECA can mimic pleomorphic adenoma, epithelial myoepithelial carcinoma, mucoepidermoid carcinoma, and polymorphous adenocarcinoma [6]. The most useful features that help distinguish MECA from a benign tumor are the histological cell pattern, the invasive pattern, and the presence of immunohistochemical myoepithelial markers such as S-100 [3].

There is no grading system for MECA [6]. It has been reported that mitotic activity, necrosis, and MECA-ex pleomorphic adenoma correlate with a worse prognosis. This tumor is aggressive, and local recurrence or distant metastasis, essentially in the lung, is the rule [6].

There is no standard treatment for MECA [6,9]. Treatment typically involves surgical resection of the tumor with or without adjuvant radiation therapy or chemotherapy, depending on the extent and aggressiveness of the cancer. Several studies have identified pleomorphic adenoma gene 1 (PLAG1) gene rearrangements and/or overexpression in approximately 53% of myoepithelial carcinomas, particularly those arising in the salivary glands [3,6]. This gene rearrangement was not present in our patient. However, we identified FGF10 amplification. There is limited research on the use of lenvatinib specifically for the treatment of MECA [9]. However, it may be considered a treatment option in certain cases, particularly if cancer has spread to other parts of the body and is not responding to other treatments [9,10].

## Conclusions

The diagnosis and treatment of myoepithelial carcinoma ex pleomorphic adenoma are not straightforward. The initial diagnosis may be delayed owing to similarities in CT imaging and cytology with pleomorphic adenoma; furthermore, the number of cases is too low to enable an adequate study of treatment approaches. As reported here, there is no standard treatment approach for this type of cancer.

Overall, myoepithelial carcinoma, ex pleomorphic adenoma, is a rare and aggressive form of cancer that requires prompt diagnosis and treatment.
